# Mapping the Role of Instructors in Canadian Post-Secondary Student Mental Health Support Systems

**DOI:** 10.1007/s11469-020-00453-3

**Published:** 2021-01-04

**Authors:** Maria Lucia DiPlacito-DeRango

**Affiliations:** Faculty of Liberal Arts & Sciences and Innovative Learning, Humber College Institute of Technology and Advanced Learning, 205 Humber College Blvd, Etobicoke, Toronto, ON M9W 5L7 Canada

**Keywords:** Post-secondary institutions, Student mental health, Support frameworks, Instructor’s role

## Abstract

Using *Recognize*, *Render*, *and Redirect* (*RRR*) (Di Placito-De Rango, *International Journal of Mental Health and Addiction* 16:284–290, 2018) as a framing organizational model, this study engaged in online document analysis to (a) locate the instructor’s position within student mental health support frameworks across Canadian colleges and universities, and (b) understand how their role is exactly defined and described. The role of instructors within student mental health support systems was detailed in 20 Canadian post-secondary institutions. Strategies to *recognize*, *render*, and *redirect* students were observed in most frameworks. For example, 45% of college and university support frameworks featured instructors engaging in compassionate narrative exchanges with students, which included instructors listening to student narratives with concern, no judgement, anti-discriminatory demeanor, and minimal interruption. Post-secondary institutions are urged to continue clearly defining and updating the role of instructors in post-secondary student mental health support frameworks.

Support for student mental health in Canadian post-secondary institutions seems more promising than it did a decade ago. In 2015, the *Okanagan Charter: An International Charter for Health Promoting Universities and Colleges* (2015) was developed, calling into action the integration and promotion of health into every sector of higher education campuses. By the end of 2020, the Ministry of Colleges and Universities (2020) is set to have invested a total of $16 million in mental health support services for students attending college and university. In collaboration with the Canadian Standards Association, the Mental Health Commission of Canada (2020a, b) developed a Canadian Standard on Psychological Health and Safety for Post-Secondary Students (PSS Standard), which is scheduled for release at some point this year. The standard will promote consistency in the implementation of “best practices” by all Canadian post-secondary institutions (Basky 2020). Pan-Canadian efforts have helped determine, expand, or improve ways that students with a mental illness or health problem are supported in post-secondary institutions.

Today, a universal design (UD) or whole systems approach to supporting student mental health seems to be the ideal path being paved in Canadian higher education settings (Canadian Association of College and University Services and Canadian Mental Health Association 2014). Put briefly, a holistic or UD approach relies on contributions from all campus parties, including administrative staff, psychologists, counsellors, and instructors, in the construction of positive and supportive learning contexts; the latter group is of particular interest in this paper, as is further explained below. Holistic approaches may become increasingly important with the rise of virtual learning delivery models (namely due to the COVID-19 pandemic) and the subsequent dismantling of shared, secure, defined, etc., environments of support.

Research on the widespread development or implementation of Canadian post-secondary student mental health support initiatives seems to focus on creating or suggesting action plans, guidelines, or roadmaps for institutional adoption (Canadian Alliance of Student Associations 2014; Canadian Association of College and University Services and Canadian Mental Health Association 2014; Mental Health Commission of Canada 2009; The Jed Foundation 2011). Few researchers have actually traced the development or evolvement of student support systems across Canadian post-secondary institutions; a surprising outcome considering the substantial efforts and pressures that circle support for student well-being in higher education settings (MacKean 2011; Yuen and Di Genova 2018)*.* MacKean’s (2011) literature and environmental scan of supportive actions in Canadian post-secondary education settings offer a very thorough understanding of trends that make up college and university mental health interventions, along with an identification of how supports can be improved. Although not as formal or nearly extensive as MacKean (2011), Yuen and Di Genova (2018) present a more current environmental scan of Canadian campus mental health strategies, outlining a timeline with links to college and university support frameworks. As commendable as the above-noted works stand, neither draw much or particular attention to the role of instructors. Student service practitioners are more commonly represented when studies decide to spotlight a specific group (Patterson and Kline 2008; Seifert and Burrow 2013).

Understanding where or how instructors are situated within student support frameworks at the university and college level raises much needed awareness towards their role in encouraging positive student mental health and well-being. Drawing attention to the institutions that effectively articulate the instructor’s role can lead to pivotal shifts in how Canadian college and university student support systems are drafted and executed. Closing the gap in support practices between live/virtual classroom, department, and policy levels can contribute to a more equitable distribution of responsibilities and overall holistic approach to addressing student mental health.

## Purpose

The purpose of this study was to (a) locate and disclose the Canadian colleges and universities that thoughtfully represent instructors within student mental health support frameworks, and (b) explain how their role is currently defined and described. Are post-secondary institutions including the role of instructors within these frameworks? How exactly do institutional strategies or systems integrate practices that instructors can employ to support student mental health? This study adopted *Recognize, Render, and Redirect (RRR)* (Di Placito-De Rango 2018) as an organizational model to frame the role of instructors in post-secondary mental health support systems. Of note, this model has not been quantitatively tested for reliability or validity. In this context, it serves as a conceptualization of organization and syntax for documenting, thinking about, and/or evaluating instructors and their support efforts with student mental health. The model is discussed further in the next section.

## Method

Document analysis was the method of research assumed. In his work, *The Essential Guide to doing your Research Project*, O’Leary (2014) states that there are three types of documents: public records, personal documents, and physical evidence. This study reviewed institutional mental health frameworks and/or policies published online, which would be considered public records—those affiliated with organizational activities (O’Leary 2014). Through his exploration of document analysis, Bowen (2009) explains that this method of qualitative research involves “evaluating documents in such a way that empirical knowledge is produced and understanding is developed” (p. 33). In other words, the purpose of this study was not to simply quantitate post-secondary student mental health support systems that broadcast the role of instructors, but rather to carefully recognize how their role is woven into the everyday strategies and practices intended to promote student well-being; to draw out the themes that define their support practices.

Research for this piece was conducted over the span of 3 months (January to March 2020). Yuen and Di Genova (2018) and MacKean’s (2011) lists of Canadian colleges and universities that identified mental health support systems were used as springboards to investigation. Then, a web search was conducted to find additional college and university student mental health support frameworks and/or policies that featured the role of instructors. The online search included varied combinations of the following terms/phrases: post*-*secondary student mental health; faculty/instructor role in supporting student mental health; faculty/instructor student support practices; faculty/instructors as frontline professionals; and college/university support for student mental health.

Frameworks and/or policies at various stages of progress were considered: currently operational, in the process of implementation, or anticipated for upcoming application. More importantly, it was critical that frameworks effectively operationalized the role of instructors in supporting student mental health. In other words, institutional frameworks that frequently and clearly articulated strategies instructors can employ—or are encouraged to employ—in support of student mental health were those reviewed. As Bowen (2009) suggests, the quality of documents is more important than quantity when engaging in document analysis. In other words, all 773 post-secondary institutions in Canada (Government of Canada 2019) may include the role of instructors in supporting student mental health, but if that inclusion entails a narrow or primitive representation, then it may limit the construction of a realistic and well-informed picture of their position. Information gathered from documents are typically organized and managed using a particular scheme—categorizing and coding data (O’Leary 2014). For this study, once the role of instructors was located within institutions’ online student mental health support systems/frameworks, their specific practices were organized thematically to mirror the *RRR* model categories.

## Recognize, Render, and Redirect (RRR) as an Organizational Model

The current study was intended to represent a timely follow-up to *Situating the Post-Secondary Instructor in a Supportive Role for the Mental Health and Well-Being of Students* (Di Placito-De Rango 2018). In this piece, I argued that the role of post-secondary instructors in supporting student mental health has been poorly defined in national, provincial, and/or institutional intervention policies/frameworks. In response, I challenged readers to (re)imagine instructors as supplemental aides in supporting the mental health of students, while at the same time, advocated for instructors by drawing attention to the multiple barriers limiting their supportive role (e.g., lack of training). I then proposed a model—*Recognize*, *Render*, *and Redirect* (*RRR*)*—*to help higher education instructors envision and promote positive student well-being (see Table 1 for an updated version of the *RRR* model). Put briefly, the model serves as a kind of toolkit for instructors; to help them *recognize* students who may be experiencing some form of distress, *render* first-line support initiatives, and *redirect* students to additional/alternative support resources on campus. With the post-secondary instructor (re)envisioned as a supplemental aide in support of student well-being, it seemed valuable to then locate and question their roles and practices within existing college/university student mental health support systems. The current study employed the *RRR* model to locate, organize, and explain the role of instructors in Canadian college/university student support frameworks.

**Table 1:** *Recognize*, *Render*, and *Redirect* model (Di Placito-De Rango 2018)

*Recognize*	Sample actions
Disclosure^1^ (via academic accommodations document or student) Signs (AWARE)^2^ (**A**cademics, **W**ell-being, **A**ttitude, **R**outine, and **E**xpected)	Instructor Receiving… • Email from student services • Email from student Instructor Observing (examples)… • Drop in grades/participation • Visible lack of hygiene • Tension with peers • Tardiness • No change (“mask of perfection”)
*Render*	Sample actions
Upon recognition Compassionate narrative exchange Needs acknowledgment and/or documentation Contact agreement Ongoing Formal academic accommodations application Support strategies application via curriculum content/expectations Support srategies application via teaching practices	Instructor (examples)… • Emailing student to inquire about needs • Asking student, “is everything ok?” • Recording student suggestions for support • Developing a progress plan Instructor (examples)… • Offering isolated testing environments • Granting task deadline extensions • Inviting a mental health guest speaker • Compartmentalizing tasks • Allowing a mental health absence pardon • Releasing course content in advance
*Redirect*	Sample actions
Crisis/emergency contact Non-Crisis/Emergency Contact	Instructor (examples)… • Calling 9-1-1 • Escorting student to campus counselling services • Emailing student advisor

^1^*Disclosure* is a form of primary recognition where the instructor is made explicitly aware of a student’s concern

^2^*Signs* is a form of secondary recognition where the instructor becomes aware of a student’s concern through observation strategies

## Results

With *RRR* as a framing organizational model, this study engaged in online document analysis to (a) locate and disclose the Canadian colleges and universities that thoughtfully represent instructors within student mental health support frameworks, and (b) explain how their role is currently defined and described. Before delving further, I would like to mention that the instructor’s role in supporting the mental health and well-being of students is not to engage in practices (e.g., diagnosing) that go beyond the scope of their expertise or employment responsibilities (Eichler and Schwartz 2010). As I have mentioned in previous work, the instructor is a frontline professional whose vantage position can be leveraged to better the psychological and learning outcomes of students.

The role of instructors within student mental health support systems was located in 20 Canadian post-secondary institutions, as revealed through their webpages (see Table 2). These institutions effectively operationalized the role of instructors in supporting student mental health. Several Canadian college and university websites (e.g., Red River College, Wilfred Laurier University, and University of Ontario Institute of Technology) were found to promote praiseworthy mental health support strategies, but exclude thoughtful consideration of the instructor’s role (such as, the signs they notice when a student is in distress and the initiatives they employ in live/virtual classrooms to improve student learning experiences). In some cases, institutions actually pointed out this particular shortcoming in their own support systems. For example, Mount Allison University (2016) and the University of Toronto (2014) both call for further clarification on instructor roles and knowledge in their respective mental health support systems (e.g., how/if instructors understand curriculum and pedagogy as impactful to student mental health). Without such clarity in support frameworks or policies, instructors can remain unsure of the responsibilities they should assume when encountering students in distress, which can leave many students unsupported (Hanlon 2012).

**Table 2:** Canadian colleges and universities that feature the role of instructors in their student mental health support systems and strategies

Camosun College Carleton University Confederation College George Brown College Humber College McMaster University Mohawk College Mount Allison University Ontario College of Art and Design (OCAD) University Queen’s University Ryerson University Simon Fraser University University of British Columbia University of Guelph University of Manitoba University of Saskatchewan University of Toronto University of Victoria York University	

Canadian colleges and universities not listed in Table 2 do not necessarily object to thoughtful consideration of the instructor’s role in student support systems. Perhaps their student mental health support frameworks may be in their infant stages of development. For example, Western University’s (2018) *Student Mental Health and Wellness Strategic Plan* clearly indicates an upcoming proposal to partner/collaborate with faculty in developing resources, participating in training, and enhancing access to support. In other cases, institutions may not be well-equipped to promote student mental health support frameworks or strategies via online communication. Interestingly, more colleges than universities lacked tactful representation of instructors, which is why Table 2 listed 15 universities and only 5 colleges. This can be due to colleges lagging slightly behind universities in hosting established student mental health frameworks/systems for whatever reason (e.g., less funding or research). Earlier this year, Colleges Ontario (2020) partnered with the Canadian Student Alliance, the Ontario Undergraduate Student Alliance, and the Council of Ontario Universities to co-develop a standard student mental health support strategy. Imaginably, persisting partnerships like these can help colleges advance in the development of student mental health support frameworks with greater consideration of the instructor’s role, like their university counterparts.

In looking more closely at how their role is defined and described, Table 3 outlines the percentages and names of Canadian colleges/universities whose institutional websites feature the roles/practices of instructors in supporting student mental health (organized through the *RRR* model). Detailed examples of some practices were also revealed.

**Table 3:** Percentages and names of Canadian post-secondary institutions (*N* = 20) that feature the roles of instructors in supporting student mental health, as per RRR categories

Role/practice *(RRR)* with examples	% of post-secondary institutions (website) featuring role/practice	Name of post-secondary institutions (website) featuring role/practice
*Recognize*		
Disclosure (via academic accommodations document or student) e.g., student disclosure of self-harm or harm to others.	55	Carleton University (2009) Confederation College (2015) George Brown College (2015) Humber College (2020) Mohawk College (2014) Queen’s University (2012) Simon Fraser University (2010) University of British Columbia (2012) University of Manitoba (2014) University of Saskatchewan (n.d.) York University (n.d.)
Signs (AWARE) (**A**cademics, **W**ell-Being, **A**ttitude, **R**outine, and **E**xpected) e.g., drop in grades, increased absence, decreased participation in live/virtual class, lack of interest, changed peer relations, and missed assignments.	40	Confederation College (2015) OCAD University (2014) Queen’s University (2012) Ryerson University (2014) Simon Fraser University (2010) University of British Columbia (2012) University of Saskatchewan (n.d.) York University (n.d.)
*Render*		
Upon recognition Compassionate narrative exchanges e.g., approaching and calmly/privately asking students open-ended questions if distress is observed, listening to student narratives with concern, no judgement, anti-discriminatory demeanor, and minimal interruption.	45	Carleton University (2009) Humber College (2020) OCAD University (2014) Ryerson University (2014) Simon Fraser University (2010) University of British Columbia (2012) University of Saskatchewan (n.d.) University of Victoria (2014–2017; n.d.) York University (n.d.)
Needs acknowledgment and/or documentation e.g., respecting privacy needs, acknowledging/validating/normalizing student thoughts/feelings/stresses, and developing a student success plan.	45	Carleton University (2009) Humber College (2020) Mohawk College (2014) OCAD University (2014) Queen’s University (2012) Ryerson University (2014) University of British Columbia (2012) University of Saskatchewan (n.d.) University of Victoria (2014-2017; n.d.)
Contact agreement e.g., establishing continued contact, observation, and/or follow-up when applicable.	15	Carleton University (2009) Simon Fraser University (2010) York University (n.d.)
Ongoing Support strategies application via curriculum content/expectations e.g., designing curriculum/course breakdown and assessments with student mental health lens in mind, course expectations clarity, workload and task weight consistency, timely feedback, flexible deadlines, adding positive rhetoric in course syllabi, and encouraging students to make connections to real life outcomes.	40	Camosun College (2016-2020) Confederation College (2015) OCAD University (2014) Queen’s University (2012) Ryerson University (2014) Simon Fraser University (2010) University of Victoria (2014-2017; n.d.) University of Waterloo (2017)
Formal academic accommodations application e.g., facilitation of standard/legal academic accommodations, extended deadlines, isolated testing locations, use of computer or other learning aids, and breaks.	10	Mohawk College (2014) University of Victoria (2014-2017)
Support strategies application via teaching practices e.g., building a trusting and non-stigmatizing live/virtual classroom, culture, and/or community, organizing ice-breakers at the start of the semester, promoting movement in the live classroom, encouraging good sleep and healthy eating, role-modelling resilience skills, and knowing the names of students.	55	Carleton University (2009) George Brown College (2015) Humber College (2020) OCAD University (2014) Ryerson University (2014) Simon Fraser University (2010) University of Manitoba (2014) University of Saskatchewan (n.d.) University of Toronto (2014) University of Victoria (2014-2017; n.d.) University of Waterloo (2017)
*Redirect*		
Crisis/emergency contact e.g., calling 9-1-1,; calling campus crisis team.	20	Humber College (2020) University of British Columbia (2012) University of Victoria (2014-2017; n.d.) York University (n.d.)
Non-crisis/emergency contact e.g., making a referral to community services, accompanying a student to campus support services, and communicating with relevant administrators	35	Carleton University (2009) Humber College (2020) McMaster University (2015) Queen’s University (2012) University of British Columbia (2012) University of Saskatchewan (n.d.) York University (n.d.)

Table 3 illustrates that the inclusion of instructors in post-secondary student mental health support systems seems to have increased over the last 5–10 years. Canadian colleges and universities are more often defining instructors as frontline professionals, acknowledging the integral part they play in supporting and promoting student well-being. Of the 20 uncovered, the following institutions featured mental health support systems with exceptional representation of instructors: Carleton University, George Brown College, Queen’s University, Simon Fraser University, University of British Columbia, University of Saskatchewan, University of Victoria, and York University. The frameworks proposed by these 8 institutions aligned best with the *RRR* organizational model categories, or in other words, captured the greatest number and variety of ways instructors can support the mental health needs of students.

Strategies to *recognize—disclosure* and *signs—*were observed in 55% and 40% of post-secondary institution mental health support frameworks respectively. Disclosure was noted to take place through both formal academic accommodation letters and student-driven revelations. Support systems displayed a wide variety of signs intended to warn instructors of students who may be experiencing distress, such as a drop in grades, increased absences, and changed peer relations, notably similar to those in *RRR*. These signs were often represented as “guidelines for instructors” to recognize students in critical and non-critical distress. It was common for support systems to list campus and community support contacts immediately following disclosure and warning signs. In the context of *RRR*, this refers to the *redirect* category.

The percentages of post-secondary institutions that detailed supportive practices for instructors to *render* were varied and unpredictable, often dependent on the type of practice. Overall, 85% of support systems listed unique, innovative, and purposeful initiatives for rendering, when compared to the *RRR* model. Specifically, 45% of college and university student support systems expressed compassionate narrative exchanges between instructors and students, as well as student needs acknowledgment and/or documentation. Examples of the former included, approaching and calmly/privately asking students open-ended questions if distress is observed, and listening to student narratives with concern, no judgement, anti-discriminatory demeanor, and minimal interruption. As for the latter, instructors acknowledging and/or documenting the needs of their students was often detailed as respecting privacy needs, as well as acknowledging/validating/normalizing student thoughts/feelings/stresses—to name a few.

Another 40% and 55% of given college/university student mental health support systems indicated that instructors render support strategies through curriculum content/expectations and teaching practices respectively. Some remarkable strategies worth noting included, workload and task weight consistency, timely feedback, adding positive rhetoric in course syllabi, organizing ice-breakers at the start of the semester, promoting movement in the live/virtual classroom, and encouraging good sleep and healthy eating. Results in the categories contact agreement and formal academic accommodations application were not as impressive. Fifteen percent of post-secondary support frameworks outlined instructor practices that denote some kind of contact or follow-up interaction between instructors and students. Likewise, only 10% of systems unveiled instructors’ application of formal academic accommodations, such as extended deadlines or isolated testing environments.

Twenty percent of the subject college and university student mental health support systems demonstrated that the role of instructors upon recognizing a student in crisis is to contact a crisis/emergency support service. Likewise, 35% of post-secondary institution frameworks suggested that instructors contact non-crisis/emergency services upon recognizing a student in a non-critical state of distress. As reflected in *RRR*, some of the non-crisis/emergency contact actions listed in the given support frameworks included, making a referral to community services, accompanying a student to campus support services, and communicating with relevant administrators*.*

## Discussion

Using document analysis and *RRR* as a framing organizational model, this study attempted to locate and disclose the Canadian colleges and universities that thoughtfully represent instructors within student mental health support frameworks, and then explain how their role is currently defined and described. It is encouraging to uncover that college and university student mental health support systems include “being aware of warning signs” and “disclosure through formal academic accommodations or student revelations” as common methods for instructors to use for recognizing students in distress. The incorporation of these practices demonstrates that the instructor’s position is critical to the recognition of students who may require support. In their work, *Faculty Views on College Student Mental Health: Implications for Retention and Student Success*, Kalkbrenner et al. (2019) reveal that instructors are comfortable and willing to identify and approach students who seem to experience some kind of distress. All of Kalkbrenner et al.’s (2019) faculty study participants described at least one warning sign they observed to identify a student with a mental health disorder, including missing class and sudden changes in behavior.

If construed through a less promising lens, suggesting that instructors are vital to or responsible for identifying warning signs or responding to disclosure efforts can put pressure on (or makes assumptions about) instructors’ knowledge and confidence in supporting student mental health. This can be in part due to continued conflicting or shifting perspectives towards the expectations of post-secondary instructors in directly supporting the emotional and/or social needs of students (Canadian Association of College and University Student Services (CACUSS) and Canadian Mental Health Association (CMHA) 2014; Eells and Rando 2010). Additionally, placing instructors as integral to or responsible for recognition efforts may not only undermine prevailing issues of mental health stigma (Corrigan et al. 2016; Martin 2010), but also ignore an institution’s responsibility in providing instructors with meaningful and accessible professional development opportunities (Margrove et al. 2014; Mowbray et al. 2010). In sum, it is valuable to learn that a considerable percentage of post-secondary institution support systems emphasize the instructor’s role in recognizing students in distress, but this does not deny the possible presence of mental health stigma limiting recognition efforts, or the need for institutions to arm instructors with the time and knowledge necessary to engage in these efforts.

Analysis of post-secondary student mental health support systems revealed that instructors *render* competent, purposeful, and sustainable support practices, after recognizing a student in distress and on an ongoing basis as well. Frameworks included an admirable amount and variety of practices (e.g., compassionate narrative exchange, needs acknowledgment and/or documentation, as well as support strategies application via curriculum content/expectations and teaching practices). Other works in this area of research represent similar customary practices exercised by instructors attempting to support their students. For example, in *Mental Health, Well-being, and Learning: Supporting Our Students in Times of Need*, Schwitzer and Vaughn (2017) assert that the first option for campus personnel who encounter a student in distress is to approach the student and engage in a thoughtful conversation about noted concerns. Additionally, with the objective of learning how curriculum can be used to increase efforts of mental health promotion, Mitchell et al. (2012) demonstrate how mental health incorporation, such as making real world connections to curriculum content, can help students build a deeper understanding of their own mental health concerns. Similarly, Mitchell et al. (2012) revealed how through collaborations with counsellors and health educators, instructors are able to infuse their course curriculum with mental health programming (e.g., helping students draw connections between academics, life, and mental health).

The above-noted practices point towards a growing trend towards widely -scoped mental health support systems development; one that calls upon “…faculty, support staff, administrators, student leaders, and students—that is, everyone on campus—to be engaged in understanding and enacting the role they play in creating a healthy campus community” (George Brown College 2015, p. 4). Put differently, the inclusion of these kinds of instructor practices strengthens the shift to universally- designed supportive learning environments. Implementing support strategies via curriculum or teaching practices can, for example, help capture even those with “masks of perfection” (i.e., show no signs of experiencing a mental illness or health problem) who can slip through the cracks. As Wynaden et al. (2014) show in their research on the silence of mental health issues in universities, student and staff survey respondents indicated that silence is infused within university campuses (e.g., due to stigma), which ultimately limits student help-seeking and formal support provision.

Despite the aforementioned efforts, percentage values for contact agreement and formal academic accommodations application were low. To recall from the *RRR* model, contact agreement refers to instructors and students agreeing to connect and/or follow-up with one another. Other works in this area of study have commonly situated contact agreement as a strategy exercised by student services or counselling/wellness departments. For example, through her exploration on the mental health needs of college students, Kitzrow (2003) indicated that counselling centers try to ensure follow-up visitations with the students they support. In this light, it can be expected that college and university student support systems exclude contact agreement as a practice for instructors to employ. Nonetheless, instructors maintaining some form of contact or follow-up with students in distress remains as a valuable practice. As Quinn et al. (2009) submit, following interviews with university students on their experiences with mental health support, many lecturers make themselves available to students for “check-ins,” academic progress updates, or any other difficulties that may arise.

Low percentage values for formal academic accommodations application were somewhat surprising, considering that literature in this subject area often places instructors as generally responsible for this practice (Murray et al. 2009; Milligan 2010; Wright and Meyer 2017). Canadian higher education settings are legally responsible, often under human rights codes/policies, to administer academic accommodations for persons with disabilities (Ontario Human Rights Commission n.d.). In response to this legal obligation, it may seem obscure or needless to include academic accommodations application in college/university student mental health support frameworks; hence, the low percentage documented. Alternatively, institutions may interpret academic accommodations as synonymous with larger student mental health support frameworks/systems. In other words, perhaps these frameworks/systems serve as what are deemed to be academic accommodations. In sum, evidence gathered from the current study suggests that post-secondary student support systems display a vast number of strategies instructors can employ to generate positive impacts in the learning environment, but would benefit from considering the role instructors can play in establishing contact agreements with students in distress, as well as substantiating greater clarity in the language and purpose surrounding academic accommodations.

As was presented in Table 3, percentage values for redirecting to crisis/emergency contact were low. It is possible that student mental health support systems are not (yet) equipped with crisis intervention or response personnel/teams; an argument forwarded by several other researchers in the given subject area (Han 2010; Myer et al. 2012). For example, through his work, *Studies on College Student Psychological Crisis Intervention System*, Han (2010) admits to this shortcoming and recommends the implementation of more “active crisis intervention electronic investigation and evaluation systems… [that] aim at different stages of development among students, and design the focus of investigation and evaluation” (p. 151). Conversely, redirection to non-crisis/emergency contact was observed more frequently in college/university student support systems, often as directly tied to instructor recognition practices. This finding is echoed in other studies (CACUSS and CMHA 2014; Froese-Germain and Riel 2012; MacKean 2011; Silverman and Glick 2010). For example, through their exploration of crisis interventions on college campuses, Silverman and Glick (2010) propose the model *QPR* (Question, Persuasion, and Referral), which essentially advises instructors to question, and persuade students to access support through referral. Likewise, Quinn et al. (2009) noted that in being “the first port of call”, instructors who acknowledge students with a mental health problem tend to refer these individuals to counselling services for the provision of more appropriate help.

It was unsurprising to observe the distinct connection between recognition and redirection considering the more straightforward and sometimes procedural nature of recognizing students in distress and then promptly redirecting them to additional campus support services. Hongjun et al.’s (2011) work on college multi-agent systems for psychological crisis prevention demonstrates how management systems and procedures can help “…stimulate human decision-making processes, so as to recognize dynamic psychological status and prevent and control crisis in time” (p. 17). In others words, the process of instructors recognizing a student in distress and redirecting them to non-crisis/emergency contacts, such as campus counselling and wellness centers, can be an automated, subconscious response. Alternatively, this may be a completely conscious act instructors adopt, whether it’s because they do not feel comfortable with or responsible for rendering support (Quinn et al. 2009; Silverman and Glick 2010; Waller et al. 2006). Automated or not, for crisis or non-crisis concerns, instructor redirection practices are essential components of college and university student support frameworks that can offer students immediate support, while at the same time, help relieve any pressures placed on instructors to carry some kind of expertise in supporting student mental health (Whitley et al. 2012).

This study was limited as it depended on public records/documents (frameworks and/or policies) published through college and university websites. Institutions with under-represented, less-sophisticated, or overall dated websites may still have student mental health support interventions in place. As such, a greater number of Canadian colleges and universities may actually have solid student mental health support strategies with clearly defined and operational roles for instructors that were missed in this study. However, what is in place is not necessarily what is in practice. To explain, it is possible that college or university instructors undertake different roles than what are promoted through larger institutional support frameworks, which can mean more or less involvement in supporting student mental health. It would not have been possible for this study to mediate the above-noted outcome, unless data was drawn directly from instructors to help better understand what they practice exactly, regardless of governing frameworks or policies. Even then, generalizability would have been difficult to achieve. Another potential drawback involves the *RRR* organizational model. As alluded to earlier, this is the first time the model has been applied, and consequently, areas for change or improvement surfaced. For example, arguably, some of the initiatives uncovered (i.e., listening to student narratives) could have been categorized as either under *Recognize* or *Render*. Perhaps this calls for a more blended approach when constructing organizational model categories.

## Conclusions

Institutional awareness has been propelled by national and global pressures to support those who battle mental illness or health problems. These pressures have led to expanding rights and legalities surrounding mental health and intervention, all of which have helped shape the instructor’s role in Canadian college and university student mental health support systems. Post-secondary institutions are urged to continue clearly defining and updating the role of instructors in supporting student mental health. In addition to collaboration, training, and funding efforts (all of which were often dramatically highlighted in the reviewed post-secondary student support systems), institutions are encouraged to continue innovating quintessential strategies that can strengthen their individualized support systems and/or solidify the position of instructors. Imaginably, institutions can establish a mental health task force, like the one at the University of Guelph (2016), who can maintain/update student support frameworks, or stimulate more nuanced, versatile, and universally-designed systems with clear instructor-driven support efforts.
